# Anomalous halogen bonds in the crystal structures of 1,2,3-tri­bromo-5-nitro­benzene and 1,3-di­bromo-2-iodo-5-nitro­benzene

**DOI:** 10.1107/S2056989015013377

**Published:** 2015-07-22

**Authors:** José A. Romero, Gerardo Aguirre Hernández, Sylvain Bernès

**Affiliations:** a Centro de Graduados e Investigación en Química, Instituto Tecnológico de Tijuana, Apdo. Postal 1166, 22510 Tijuana, B.C., Mexico; b Instituto de Física, Benemérita Universidad Autónoma de Puebla, Av. San Claudio y 18 Sur, 72570 Puebla, Pue., Mexico

**Keywords:** crystal structure, polyhalogenated benzene, halogen bond, bromine, iodine

## Abstract

The title trihalogenated nitro­benzene derivatives, C_6_H_2_Br_3_NO_2_ and C_6_H_2_Br_2_INO_2_, crystallize in triclinic and monoclinic cells, respectively, with two mol­ecules per asymmetric unit in each case. The asymmetric unit of the tri­bromo compound features a polarized Br^δ+^⋯Br^δ-^ inter­molecular halogen bond. After substitution of the Br atom in the *para* position with respect to the nitro group, the network of *X*⋯*X* halogen contacts is reorganized. Two inter­molecular polarized halogen bonds are then observed, which present the uncommon polarization Br^δ+^⋯I^δ-^: the more electronegative site (Br) behaves as a donor and the less electronegative site (I) as an acceptor for the charge transfer.

## Abstract 2

Two halogenated nitro­benzene derivatives have been characterized. The substitution of a Br substituent by an I atom modifies the network of halogen bonds, and gives rise to the formation of non-classical Br^δ+^⋯I^δ-^ bonds.

## Chemical context   

Within the large class of non-covalent inter­actions studied in chemical crystallography, halogen bonds are of special inter­est in crystal engineering. The stabilizing inter­action between a halogen atom and a Lewis base, *X*⋯B, shares many aspects with classical hydrogen bonds, but is more directional. On the other hand, halogen contacts *X*⋯*X* are more difficult to conceptualize (Wang *et al.*, 2014 ▸), for instance because the charge transfer in the Br⋯Br contact is not as obvious as in hydrogen bonds. Evidence supporting the importance of this topic is the recent organization of an inter­national meeting dedicated to halogen bonding (Erdelyi, 2014 ▸).
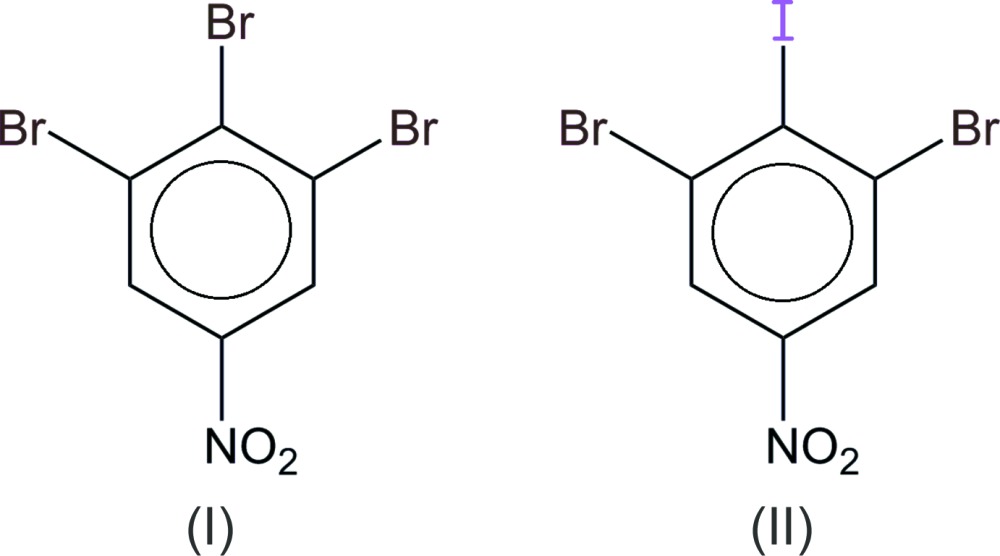


In this context, we are engaged in the synthesis and structural characterization of a series of halogen-substituted nitro­benzenes. The present communication describes two closely related compounds in the series, which differ only by the halogen atom substituting at the ring position *para* to the nitro group. Despite the small chemical modification, the resulting crystal structures are very different, as a consequence of a different network of halogen bonds.

## Structural commentary   

**Figure 1:**
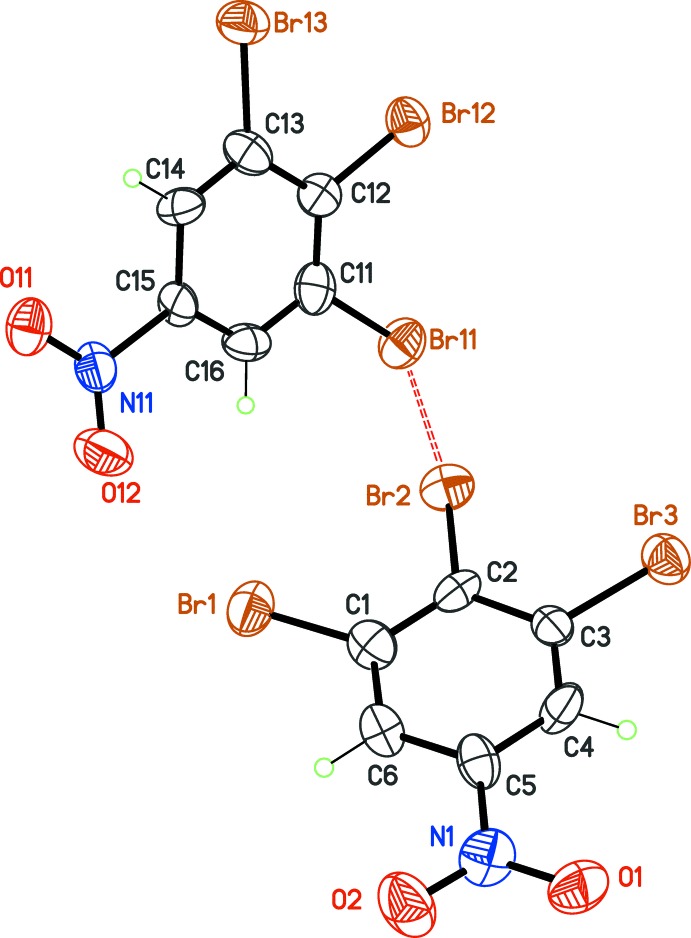
The asymmetric unit of (I), with displacement ellipsoids at the 30% probability level. The dashed bond connecting the independent mol­ecules is a type-II halogen bond.

**Figure 2:**
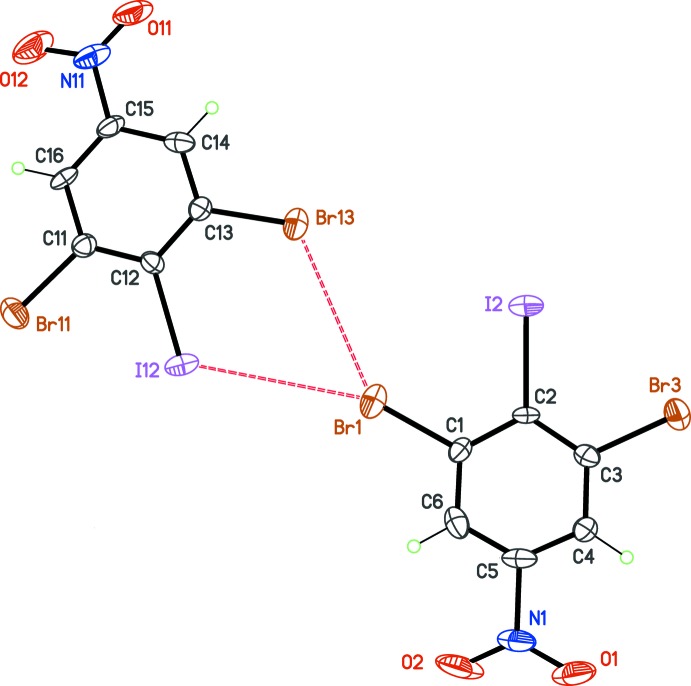
The asymmetric unit of (II), with displacement ellipsoids at the 30% probability level. The dashed bonds connecting the independent mol­ecules are halogen contacts.

Both compounds crystallize with two mol­ecules in the asymmetric unit, but in different space groups. The tri­bromo derivative, (I, Fig. 1 ▸), is a *P* 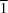 crystal isomorphous to the chloro analogue (Bhar *et al.*, 1995 ▸), although the unit-cell parameters are significantly larger for (I) compared to the chloro compound: the cell volume is increased by more than 7%. In the present work, we retained the Niggli reduced triclinic cell (*a* < *b* < *c*), while Bhar *et al.* used a non-reduced cell. Moreover, the asymmetric unit content was defined in order to emphasize the strongest Br⋯Br bond in (I). The bromo-iodo derivative (II, Fig. 2 ▸) crystallizes in the monoclinic system and, in that case, the standard setting was used for space group *P*2_1_/*c*.

The C—halogen bond lengths are as expected. In (I), C—Br distances are in the range 1.821 (12)–1.886 (11) Å, slightly shorter than C—Br bond lengths observed in hexa­bromo­benzene, 1.881 Å (*T* = 100 K; Reddy *et al.*, 2006 ▸) or 1.871 Å (synchrotron study, *T* = 100 K; Brezgunova *et al.*, 2012 ▸). In (II), C—Br bond lengths are longer, 1.875 (13) to 1.895 (14) Å, while the C—I bond lengths, 2.088 (12) and 2.074 (14) Å, may be compared to bonds in hexa­iodo­benzene, 2.109 Å (*T* = 100 K; Ghosh *et al.*, 2007 ▸) or 1,2,3-tri­iodo­benzene, 2.090 Å (*T* = 223 K, Novak & Li, 2007 ▸). Indeed, differences in bond lengths between perhalogenated and trihalogenated derivatives are within experimental errors, and the substitution of the 5-position by the nitro electron-withdrawing group in (I) and (II) has probably little influence on these bonds.

The important feature in these halogenated mol­ecules is rather the possibility of steric repulsion between vicinal halogen atoms, which is related to the reduction of endocyclic angles. Regarding this point, it is worth reading the *Acta E* article about 1,2,3-tri­iodo­benzene (Novak & Li, 2007 ▸). As in polyiodo derivatives, intra­molecular steric crowding between the halogen atoms in (I) and (II) is offset by benzene ring distortion. As a consequence, the C1—C2—C3 and equivalent C11—C12—C13 angles are systematically less than 120°: 116.2 (11) and 118.8 (13)° in (I); 118.1 (12) and 117.3 (13)° in (II). Again, the nitro group has little influence on intra­molecular halogen⋯halogen contacts. For instance, in 1,3-di­bromo-2-iodo­benzene, the C1—C2—C3 angle is 118.0° (Schmidbaur *et al.*, 2004 ▸), very close to that observed in (II), which presents the same halogen substitution.

The 5-nitro substituent is almost conjugated with the benzene nucleus in (I): the dihedral angle between the NO_2_ plane and the benzene ring is 6(2) and 1(2)° for each independent mol­ecule. For (II), twisting of the NO_2_ groups is more significant, with dihedral angles of 10 (1) and 7(1)°. This near planar conformation is identical to that observed for 1,2,3-tri­chloro-5-nitro­benzene (Bhar *et al.*, 1995 ▸), but contrasts with the twisted conformation observed in perhalogenated nitro­benzene derivatives: penta­chloro­nitro­benzene (twist angle of NO_2_: 62°; Tanaka *et al.*, 1974 ▸) and 1-bromo-2,3,5,6-tetra­fluoro-4-nitro­benzene (twist angle of NO_2_: 41.7 (3)°; Stein *et al.*, 2011 ▸). It thus seems clear that twisting of the nitro group with respect to the benzene ring in nitro­benzene derivatives is a direct consequence of intra­molecular crowding with *ortho* substituents. For 1,2,3-halogenated-5-nitro­benzenes such as (I) and (II), a planar conformation should be expected as a rule.

## Supra­molecular features   

**Figure 3:**
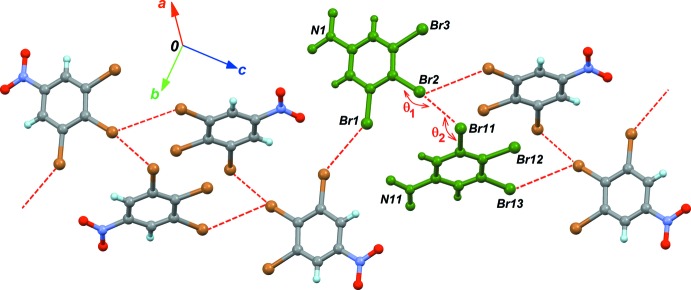
Part of the crystal structure of (I), emphasizing the halogen bonds (dashed lines). The green mol­ecules correspond to the asymmetric unit.

The crystal structures are directed by inter­molecular weak halogen bonds, also known as type-II inter­actions in the Desiraju classification scheme (Reddy *et al.*, 2006 ▸). Such a bond is present in the asymmetric unit of (I), between Br2 and Br11 (Fig. 3 ▸). The type-II arrangement is characterized by angles *θ*_1_ = C2—Br2⋯Br11 and *θ*_2_ = C11—Br11⋯Br2, which should be close to 180 and 90°, respectively. For (I), observed angles are *θ*_1_ = 165.2 (5)° and *θ*_2_ = 82.3 (5)°. The crystal packing thus polarizes the involved halogen atoms, forming the halogen bond Br2^δ+^⋯Br11^δ-^. This dimolecular polar unit is connected *via* inversion centers to neighboring units in the cell, forming C—H⋯Br hydrogen bonds, and O⋯Br contacts. This packing motif induces secondary halogen⋯halogen contacts, which are clearly unpolarized. These type-I inter­actions are characterized by angles *θ*_1_ ≃ *θ*_2_ (Table 1 ▸, entries 2 and 3) and display larger Br⋯Br separations compared to the polarized halogen bond (entry 1), in which electrostatic forces bring the atoms into close contact.

**Figure 4:**
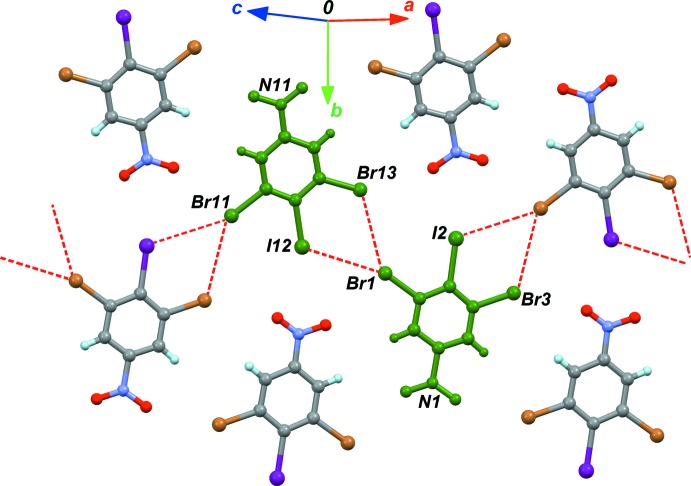
Part of the crystal structure of (II), emphasizing the halogen bonds (dashed lines). The green mol­ecules correspond to the asymmetric unit.

The substitution of one Br atom by I, to form crystal (II), changes dramatically the packing structure, affording a more complex network of halogen contacts (Fig. 4 ▸ and Table 2 ▸). Within the asymmetric unit, the type-II polarized contact is Br1⋯I12 (Table 2 ▸, entry 1). However, *θ* angles for this bond deviate from ideal values, and, surprisingly, the bond is polarized in the wrong way, Br^δ+^⋯I^δ-^. The opposite polarization was expected for this bond, due to the lower electronegativity and higher polarizability of iodine compared to bromine. The other significant contact observed in the asymmetric unit is a Br⋯Br unpolarized contact. The network of halogen bonds is expanded in the [100] direction by Br11, which gives a bifurcated contact with I2 and Br3 (Table 2 ▸, entries 2 and 4). One contact is polarized, with the polarization, once again, oriented in the unexpected way, I2^δ-^⋯Br11^δ+^. These anomalous halogen bonds are not present in other mixed halogen derivatives. Indeed, in 1,3-di­bromo-2-iodo­benzene (Schmidbaur *et al.*, 2004 ▸), the iodine atom is not engaged in halogen bonding.

## Database survey   

The current release of the CSD (Version 5.36 with all updates; Groom & Allen, 2014 ▸), contains many structures of halogen-substituted nitro­benzene, with Cl (*e.g*. Bhar *et al.*, 1995 ▸; Tanaka *et al.*, 1974 ▸), Br (*e.g.* Olaru *et al.*, 2014 ▸), and I (Thalladi *et al.*, 1996 ▸). This series is completed with nitro­phenol deriv­atives, for example 2,3-di­fluoro-4-iodo-6-nitro­phenol (Francke *et al.*, 2010 ▸). Structures of penta­chloro­phenol (Brezgunova *et al.*, 2012 ▸) and penta­bromo­phenol (Betz *et al.*, 2008 ▸; Brezgunova *et al.*, 2012 ▸) are also available.

Regarding poly- and per-halogenated benzene structures, an impressive series of 23 compounds has been described, including Cl, Br, I and Me as substituents, generating a variety of mol­ecular symmetries (Reddy *et al.*, 2006 ▸). The structure of *D*_6h_-perhalogenated benzene has been reported with F (Shorafa *et al.*, 2009 ▸), Cl (Brown & Strydom, 1974 ▸; Reddy *et al.*, 2006 ▸), Br (Baharie & Pawley, 1979 ▸; Reddy *et al.*, 2006 ▸; Brezgunova *et al.*, 2012 ▸) and I (Ghosh *et al.*, 2007 ▸). The former is a *Z*′ = 2 crystal, while others are *Z*′=1 crystals.

## Synthesis and crystallization   

**Figure 5:**
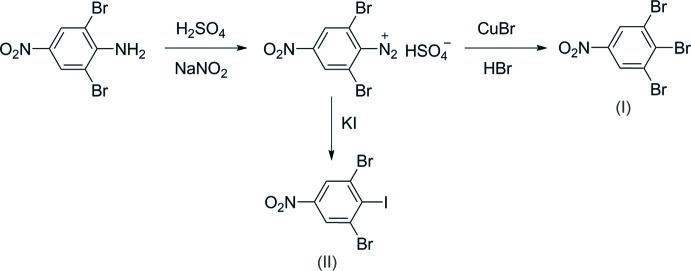
Synthetic scheme for (I) and (II).

Compounds (I) and (II) were synthesized from 2,6-di­bromo-4-nitro­aniline (Bryant *et al.*, 1998 ▸), as depicted in Fig. 5 ▸.

**Synthesis of (I)**. A solution of 2,6-di­bromo-4-nitro­aniline (1.0 g, 3.38 mmol) in acetic acid (3 ml) was cooled to 278 K, and concentrated H_2_SO_4_ (7 ml) was carefully added under stirring. While ensuring that the temperature was still below 278 K, NaNO_2_ (0.708 g, 10.26 mmol) was added in one batch. The reaction was stirred at this temperature for 2 h to afford the diazo­nium salt. An aqueous solution (17.67 ml) of CuBr (4.95 g, 34.54 mmol) and 47% HBr (17.67 ml) was warmed to 343 K, and the diazo­tization solution previously prepared was added in one batch with stirring. The mixture was kept at 343 K for 1 h, and then left to cool overnight. The reaction was neutralized with NaOH and extracted with CH_2_Cl_2_ (3 × 30 ml). The resulting solution was concentrated under vacuum and the crude material was purified by flash chromatography (petroleum ether/CH_2_Cl_2_ 8/2, *R*_f_ = 0.49) to give (I). Crystals were obtained by slow evaporation of a methanol/ethyl ether solution (yield: 0.952 g, 2.65 mmol, 78%). m.p. 380–382 K. IR (KBr, cm^−1^): 3090 (Ar—H); 1583 (C=C); 1526, 1342 (N=O); 738 (C—Br). ^1^H-NMR (600 MHz, CDCl_3_): δ 8.43 (*s*, H-4, H-6). ^13^C-NMR (150 MHz, CDCl_3_): δ 146.8, 135.7, 127.0, 126.9, 126.8. EIMS *m/z*: [*M* ^+^] 357 (34), [*M* ^+^+2] 359 (7), [*M* ^+^+4] 361 (100), [*M* ^+^+6] 363 (36) [*M* ^+^-NO_2_] 311 (12).

**Synthesis of (II)**. A solution of 2,6-di­bromo-4-nitro­aniline (1.0 g, 3.38 mmol) in acetic acid (3 ml) was cooled to 278 K in an ice-salt bath, and concentrated H_2_SO_4_ (3 ml) was carefully added under stirring. While ensuring that the temperature was still below 278 K, NaNO_2_ (0.242 g, 3.516 mmol) was added in one batch. The reaction was stirred at this temperature for 30 min to afford the diazo­nium salt. An aqueous solution (10 ml) of KI (5.635 g, 33.95 mmol) was prepared, and the diazo­tization solution previously prepared was added in one batch. The mixture was then further stirred for 1 h. The reaction was neutralized with NaOH, extracted with CH_2_Cl_2_ (3 × 30 ml), and concentrated under vacuum. The crude material was purified by flash chromatography (petroleum ether/CH_2_Cl_2_ 4/1, *R*_f_ = 0.31) to give (II). Crystals were obtained by slow evaporation of an acetone/methanol/CH_2_Cl_2_ solution (yield: 1.21 g, 2.98 mmol, 88%). m.p. 415–417 K. IR (KBr, cm^−1^): 3010 (Ar—H); 1620, 1516 (C=C); 1336 (N=O). ^1^H-NMR (600 MHz, CDCl_3_): δ 8.38 (*s*, H-4, H-6). ^13^C-NMR (150 MHz, CDCl_3_): δ 146.1, 142.4, 127.4, 124.1. EIMS *m/z*: [*M* ^+^] 405 (42), [*M* ^+^+2] 407 (100), [*M* ^+^+4] 409 (48).

## Refinement   

Crystal data, data collection and structure refinement details for (I) and (II) are summarized in Table 3 ▸. The absorption correction for (I) was challenging, and eventually carried out by applying *DIFABS* on the complete isotropic model (Walker & Stuart, 1983 ▸). In the case of (II), measured ψ-scans were used. H atoms were refined as riding to their carrier C atoms, with C—H bond lengths fixed at 0.93 Å and with *U*_iso_(H) = 1.2*U*_eq_(carrier atom).

## Supplementary Material

Crystal structure: contains datablock(s) I, II, global. DOI: 10.1107/S2056989015013377/hb7459sup1.cif

Structure factors: contains datablock(s) I. DOI: 10.1107/S2056989015013377/hb7459Isup2.hkl

Structure factors: contains datablock(s) II. DOI: 10.1107/S2056989015013377/hb7459IIsup3.hkl

Supporting information file. DOI: 10.1107/S2056989015013377/hb7459Isup4.cml

Supporting information file. DOI: 10.1107/S2056989015013377/hb7459IIsup5.cml

CCDC references: 1412444, 1412445

Additional supporting information:  crystallographic information; 3D view; checkCIF report

## Figures and Tables

**Table 1:** Halogen-bond geometry (Å, °) for (I)

*X*_1_⋯*X*_2_	*d*	θ_1_	θ_2_	bond type
Br2⋯Br11	3.642 (3)	165.2 (5)	82.3 (5)	II-polarized
Br1⋯Br1^i^	3.731 (4)	133.3 (4)	133.3 (4)	I-unpolarized
Br2⋯Br13^ii^	3.781 (3)	126.8 (4)	129.6 (4)	I-unpolarized

Notes: *d* = separation *X*_1_⋯*X*_2_; θ_1_ = angle C—*X*_1_⋯*X*_2_; θ_2_ = angle *X*_1_⋯*X*_2_—C. For halogen bond types, see: Reddy *et al.* (2006 ▸). Symmetry codes: (i) −*x*, 1 − *y*, −*z*; (ii) −*x*, −*y*, 1 − *z*.

**Table 2:** Halogen-bond geometry (Å, °) for (II)

*X*_1_⋯*X*_2_	*d*	θ_1_	θ_2_	bond type
Br1⋯I12	3.813 (2)	161.2 (4)	117.2 (4)	II-polarized
I2⋯Br11^i^	3.893 (2)	116.6 (4)	161.8 (4)	II-polarized
Br1⋯Br13	3.787 (2)	142.8 (4)	122.9 (4)	I-unpolarized
Br11⋯Br3^ii^	3.858 (2)	143.9 (4)	124.4 (4)	I-unpolarized

Notes: *d* = separation *X*_1_⋯*X*_2_; θ_1_ = angle C—*X*_1_⋯*X*_2_; θ_2_ = angle *X*_1_⋯*X*_2_—C. For halogen bond types, see: Reddy *et al.* (2006 ▸). Symmetry codes: (i) 1 + *x*, *y*, *z*; (ii) −1 + *x*, *y*, *z*.

**Table 3:** Experimental details

	(I)	(II)
Crystal data		
Chemical formula	C_6_H_2_Br_3_NO_2_	C_6_H_2_Br_2_INO_2_
*M*_r_	359.82	406.81
Crystal system, space group	Triclinic, *P* 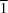	Monoclinic, *P*2_1_/*c*
Temperature (K)	298	298
*a*, *b*, *c* (Å)	7.641 (5), 8.040 (5), 14.917 (6)	13.548 (3), 20.037 (3), 9.123 (2)
α, β, γ (°)	83.91 (3), 79.86 (4), 81.49 (4)	90, 130.37 (2), 90
*V* (Å^3^)	889.2 (8)	1886.8 (8)
*Z*	4	8
Radiation type	Mo *K*α	Mo *K*α
μ (mm^−1^)	13.57	11.82
Crystal size (mm)	0.42 × 0.40 × 0.30	0.50 × 0.22 × 0.12

Data collection		
Diffractometer	Bruker P4	Bruker P4
Absorption correction	Part of the refinement model (Δ*F*) (Walker & Stuart, 1983 ▸)	ψ scan (*XSCANS*; Bruker, 1997 ▸)
*T*_min_, *T*_max_	0.0002, 0.001	0.429, 0.988
No. of measured, independent and observed [*I* > 2σ(*I*)] reflections	6070, 3141, 1503	5716, 5407, 1968
*R*_int_	0.120	0.058
(sin θ/λ)_max_ (Å^−1^)	0.596	0.703

Refinement		
*R*[*F* ^2^ > 2σ(*F* ^2^)], *wR*(*F* ^2^), *S*	0.066, 0.196, 1.47	0.061, 0.153, 0.95
No. of reflections	3141	5407
No. of parameters	218	218
H-atom treatment	H-atom parameters constrained	H-atom parameters constrained
Δρ_max_, Δρ_min_ (e Å^−3^)	0.79, −1.00	0.84, −0.84

Computer programs: *XSCANS* (Bruker, 1997 ▸), *SHELXS2014* (Sheldrick, 2008 ▸), *SHELXL2014* (Sheldrick, 2015 ▸) and *Mercury* (Macrae *et al.*, 2008 ▸).

## supplementary crystallographic information

### (I) 1,2,3-Tribromo-5-nitrobenzene Crystal data

**Table:**

C_6_H_2_Br_3_NO_2_	*F*(000) = 664
*M**_r_* = 359.82	*D*_x_ = 2.688 Mg m^−^^3^
Triclinic, *P*1	Melting point: 380 K
*a* = 7.641 (5) Å	Mo *K*α radiation, λ = 0.71073 Å
*b* = 8.040 (5) Å	Cell parameters from 48 reflections
*c* = 14.917 (6) Å	θ = 4.8–12.4°
α = 83.91 (3)°	µ = 13.57 mm^−^^1^
β = 79.86 (4)°	*T* = 298 K
γ = 81.49 (4)°	Irregular, colourless
*V* = 889.2 (8) Å^3^	0.42 × 0.40 × 0.30 mm
*Z* = 4	

### (I) 1,2,3-Tribromo-5-nitrobenzene Data collection

**Table:**

Bruker P4 diffractometer	1503 reflections with *I* > 2σ(*I*)
Radiation source: fine-focus sealed tube	*R*_int_ = 0.120
Graphite monochromator	θ_max_ = 25.1°, θ_min_ = 2.6°
ω scans	*h* = −8→9
Absorption correction: part of the refinement model (Δ*F*) (Walker & Stuart, 1983)	*k* = −9→9
*T*_min_ = 0.0002, *T*_max_ = 0.001	*l* = 0→17
6070 measured reflections	3 standard reflections every 97 reflections
3141 independent reflections	intensity decay: 1%

### (I) 1,2,3-Tribromo-5-nitrobenzene Refinement

**Table:**

Refinement on *F*^2^	Secondary atom site location: difference Fourier map
Least-squares matrix: full	Hydrogen site location: inferred from neighbouring sites
*R*[*F*^2^ > 2σ(*F*^2^)] = 0.066	H-atom parameters constrained
*wR*(*F*^2^) = 0.196	*w* = 1/[σ^2^(*F*_o_^2^) + (0.050*P*)^2^] where *P* = (*F*_o_^2^ + 2*F*_c_^2^)/3
*S* = 1.47	(Δ/σ)_max_ < 0.001
3141 reflections	Δρ_max_ = 0.79 e Å^−^^3^
218 parameters	Δρ_min_ = −1.00 e Å^−^^3^
0 restraints	Extinction correction: SHELXL2014 (Sheldrick, 2015), Fc^*^=kFc[1+0.001xFc^2^λ^3^/sin(2θ)]^-1/4^
0 constraints	Extinction coefficient: 0.0063 (12)
Primary atom site location: structure-invariant direct methods	

### (I) 1,2,3-Tribromo-5-nitrobenzene Fractional atomic coordinates and isotropic or equivalent isotropic displacement parameters (Å^2^)

**Table:**

	*x*	*y*	*z*	*U*_iso_*/*U*_eq_	
Br1	0.1438 (2)	0.3194 (2)	0.05082 (13)	0.0863 (6)	
Br2	0.2958 (2)	0.0254 (2)	0.20089 (11)	0.0855 (6)	
Br3	0.4312 (2)	−0.3604 (2)	0.13778 (10)	0.0791 (5)	
C1	0.2177 (18)	0.1018 (19)	0.0215 (9)	0.070 (4)	
C2	0.2831 (17)	−0.0235 (17)	0.0860 (9)	0.063 (3)	
C3	0.3360 (14)	−0.1910 (16)	0.0555 (8)	0.056 (3)	
C4	0.3227 (18)	−0.2259 (19)	−0.0296 (9)	0.069 (4)	
H4	0.3573	−0.3355	−0.0465	0.083*	
C5	0.2615 (17)	−0.1072 (16)	−0.0895 (10)	0.064 (3)	
C6	0.2067 (16)	0.0562 (16)	−0.0661 (9)	0.059 (3)	
H6	0.1619	0.1373	−0.1085	0.071*	
N1	0.2502 (16)	−0.1453 (18)	−0.1809 (8)	0.075 (3)	
O1	0.2967 (16)	−0.2925 (16)	−0.2012 (7)	0.094 (3)	
O2	0.1923 (18)	−0.0342 (15)	−0.2318 (8)	0.105 (4)	
Br11	0.3943 (2)	0.2012 (2)	0.39885 (13)	0.0891 (6)	
Br12	0.1013 (2)	0.1170 (2)	0.58586 (10)	0.0795 (6)	
Br13	−0.3231 (2)	0.2845 (2)	0.59130 (11)	0.0865 (6)	
C11	0.151 (2)	0.2912 (18)	0.4092 (12)	0.077 (4)	
C12	0.0303 (18)	0.2505 (18)	0.4860 (10)	0.064 (3)	
C13	−0.150 (2)	0.323 (2)	0.4913 (9)	0.073 (4)	
C14	−0.2002 (19)	0.4188 (19)	0.4208 (9)	0.070 (4)	
H14	−0.3207	0.4619	0.4232	0.084*	
C15	−0.079 (2)	0.4583 (18)	0.3427 (8)	0.068 (4)	
C16	0.0943 (19)	0.3923 (18)	0.3375 (9)	0.068 (4)	
H16	0.1756	0.4155	0.2850	0.082*	
N11	−0.1327 (18)	0.5801 (16)	0.2644 (10)	0.076 (3)	
O11	−0.2882 (14)	0.6432 (13)	0.2757 (7)	0.083 (3)	
O12	−0.0224 (17)	0.5952 (18)	0.1956 (8)	0.110 (4)	

### (I) 1,2,3-Tribromo-5-nitrobenzene Atomic displacement parameters (Å^2^)

**Table:**

	*U*^11^	*U*^22^	*U*^33^	*U*^12^	*U*^13^	*U*^23^
Br1	0.0776 (11)	0.0671 (10)	0.1094 (12)	−0.0047 (8)	−0.0022 (9)	−0.0140 (8)
Br2	0.0876 (11)	0.0974 (13)	0.0716 (9)	−0.0124 (9)	−0.0075 (8)	−0.0164 (8)
Br3	0.0765 (10)	0.0789 (11)	0.0793 (10)	−0.0043 (8)	−0.0186 (8)	0.0070 (8)
C1	0.062 (8)	0.078 (10)	0.072 (9)	−0.013 (7)	−0.022 (7)	0.001 (7)
C2	0.058 (8)	0.068 (9)	0.062 (8)	−0.006 (7)	0.004 (6)	−0.026 (7)
C3	0.034 (6)	0.064 (8)	0.060 (7)	−0.001 (6)	0.001 (6)	0.012 (6)
C4	0.063 (8)	0.072 (9)	0.071 (9)	0.022 (7)	−0.018 (7)	−0.030 (7)
C5	0.056 (8)	0.051 (8)	0.087 (10)	−0.015 (6)	−0.021 (7)	0.009 (7)
C6	0.062 (8)	0.052 (8)	0.073 (8)	−0.019 (6)	−0.033 (7)	0.003 (6)
N1	0.079 (8)	0.081 (9)	0.080 (8)	−0.016 (7)	−0.036 (7)	−0.026 (7)
O1	0.119 (9)	0.095 (9)	0.071 (6)	−0.005 (7)	−0.017 (6)	−0.032 (6)
O2	0.151 (11)	0.087 (9)	0.089 (7)	−0.010 (8)	−0.061 (8)	0.004 (6)
Br11	0.0644 (10)	0.0993 (13)	0.1026 (12)	0.0018 (9)	−0.0185 (9)	−0.0137 (10)
Br12	0.0955 (12)	0.0701 (10)	0.0769 (9)	−0.0090 (8)	−0.0294 (9)	−0.0017 (7)
Br13	0.0785 (11)	0.0945 (13)	0.0808 (10)	−0.0202 (9)	0.0012 (8)	0.0067 (9)
C11	0.084 (10)	0.050 (8)	0.102 (11)	−0.013 (7)	−0.021 (9)	−0.015 (8)
C12	0.060 (8)	0.062 (8)	0.073 (9)	−0.018 (7)	−0.012 (8)	−0.008 (7)
C13	0.081 (10)	0.082 (10)	0.060 (8)	−0.020 (8)	−0.024 (7)	0.009 (7)
C14	0.056 (8)	0.080 (10)	0.065 (8)	−0.010 (7)	0.011 (7)	−0.007 (7)
C15	0.088 (10)	0.077 (10)	0.040 (6)	0.019 (8)	−0.034 (7)	−0.004 (6)
C16	0.070 (9)	0.072 (9)	0.062 (8)	−0.028 (8)	0.006 (7)	−0.003 (7)
N11	0.067 (8)	0.065 (8)	0.093 (10)	−0.005 (6)	−0.017 (7)	0.011 (7)
O11	0.074 (7)	0.079 (7)	0.099 (7)	0.003 (6)	−0.032 (6)	−0.014 (6)
O12	0.097 (8)	0.144 (12)	0.077 (7)	−0.012 (8)	−0.011 (7)	0.028 (7)

### (I) 1,2,3-Tribromo-5-nitrobenzene Geometric parameters (Å, º)

**Table:**

Br1—C1	1.831 (15)	Br11—C11	1.877 (15)
Br2—C2	1.821 (12)	Br12—C12	1.854 (14)
Br3—C3	1.886 (11)	Br13—C13	1.842 (15)
C1—C6	1.415 (18)	C11—C16	1.368 (19)
C1—C2	1.416 (18)	C11—C12	1.38 (2)
C2—C3	1.445 (18)	C12—C13	1.410 (19)
C3—C4	1.353 (17)	C13—C14	1.313 (18)
C4—C5	1.328 (17)	C14—C15	1.39 (2)
C4—H4	0.9300	C14—H14	0.9300
C5—C6	1.381 (19)	C15—C16	1.347 (19)
C5—N1	1.448 (18)	C15—N11	1.515 (16)
C6—H6	0.9300	C16—H16	0.9300
N1—O2	1.194 (15)	N11—O11	1.211 (15)
N1—O1	1.238 (16)	N11—O12	1.216 (16)
			
C6—C1—C2	119.0 (13)	C16—C11—C12	120.4 (14)
C6—C1—Br1	119.9 (9)	C16—C11—Br11	118.6 (13)
C2—C1—Br1	121.0 (10)	C12—C11—Br11	120.9 (11)
C1—C2—C3	116.2 (11)	C11—C12—C13	118.8 (13)
C1—C2—Br2	121.3 (10)	C11—C12—Br12	122.1 (10)
C3—C2—Br2	122.5 (9)	C13—C12—Br12	118.9 (10)
C4—C3—C2	121.8 (11)	C14—C13—C12	118.8 (14)
C4—C3—Br3	120.6 (10)	C14—C13—Br13	118.0 (11)
C2—C3—Br3	117.6 (9)	C12—C13—Br13	123.1 (10)
C5—C4—C3	121.5 (13)	C13—C14—C15	122.5 (13)
C5—C4—H4	119.3	C13—C14—H14	118.8
C3—C4—H4	119.3	C15—C14—H14	118.8
C4—C5—C6	120.7 (13)	C16—C15—C14	119.2 (11)
C4—C5—N1	121.1 (13)	C16—C15—N11	117.9 (13)
C6—C5—N1	118.2 (11)	C14—C15—N11	122.8 (12)
C5—C6—C1	120.8 (11)	C15—C16—C11	120.0 (14)
C5—C6—H6	119.6	C15—C16—H16	120.0
C1—C6—H6	119.6	C11—C16—H16	120.0
O2—N1—O1	123.6 (12)	O11—N11—O12	127.0 (13)
O2—N1—C5	118.3 (13)	O11—N11—C15	114.7 (13)
O1—N1—C5	118.1 (12)	O12—N11—C15	118.1 (12)
			
C6—C1—C2—C3	0.6 (19)	C16—C11—C12—C13	−4 (2)
Br1—C1—C2—C3	179.5 (9)	Br11—C11—C12—C13	179.1 (11)
C6—C1—C2—Br2	−178.5 (10)	C16—C11—C12—Br12	−178.9 (11)
Br1—C1—C2—Br2	0.3 (16)	Br11—C11—C12—Br12	4.0 (17)
C1—C2—C3—C4	−0.3 (19)	C11—C12—C13—C14	4 (2)
Br2—C2—C3—C4	178.8 (11)	Br12—C12—C13—C14	179.4 (12)
C1—C2—C3—Br3	178.0 (9)	C11—C12—C13—Br13	−178.0 (11)
Br2—C2—C3—Br3	−2.8 (14)	Br12—C12—C13—Br13	−2.7 (18)
C2—C3—C4—C5	1 (2)	C12—C13—C14—C15	−3 (2)
Br3—C3—C4—C5	−177.8 (11)	Br13—C13—C14—C15	178.7 (12)
C3—C4—C5—C6	−1 (2)	C13—C14—C15—C16	2 (2)
C3—C4—C5—N1	179.1 (13)	C13—C14—C15—N11	−174.8 (15)
C4—C5—C6—C1	1 (2)	C14—C15—C16—C11	−2 (2)
N1—C5—C6—C1	−178.8 (12)	N11—C15—C16—C11	175.4 (13)
C2—C1—C6—C5	−1 (2)	C12—C11—C16—C15	3 (2)
Br1—C1—C6—C5	−180.0 (10)	Br11—C11—C16—C15	179.8 (11)
C4—C5—N1—O2	179.2 (14)	C16—C15—N11—O11	−175.5 (13)
C6—C5—N1—O2	−1 (2)	C14—C15—N11—O11	1 (2)
C4—C5—N1—O1	1 (2)	C16—C15—N11—O12	10 (2)
C6—C5—N1—O1	−178.8 (13)	C14—C15—N11—O12	−173.5 (15)

### (II) 1,3-Dibromo-2-iodo-5-nitrobenzene Crystal data

**Table:**

C_6_H_2_Br_2_INO_2_	*D*_x_ = 2.864 Mg m^−^^3^
*M**_r_* = 406.81	Melting point: 415 K
Monoclinic, *P*2_1_/*c*	Mo *K*α radiation, λ = 0.71073 Å
*a* = 13.548 (3) Å	Cell parameters from 43 reflections
*b* = 20.037 (3) Å	θ = 5.7–12.5°
*c* = 9.123 (2) Å	µ = 11.82 mm^−^^1^
β = 130.37 (2)°	*T* = 298 K
*V* = 1886.8 (8) Å^3^	Prism, brown
*Z* = 8	0.50 × 0.22 × 0.12 mm
*F*(000) = 1472	

### (II) 1,3-Dibromo-2-iodo-5-nitrobenzene Data collection

**Table:**

Bruker P4 diffractometer	1968 reflections with *I* > 2σ(*I*)
Radiation source: fine-focus sealed tube	*R*_int_ = 0.058
Graphite monochromator	θ_max_ = 30.0°, θ_min_ = 2.2°
2θ/ω scans	*h* = −14→19
Absorption correction: ψ scan (XSCANS; Bruker, 1997)	*k* = 0→28
*T*_min_ = 0.429, *T*_max_ = 0.988	*l* = −12→0
5716 measured reflections	3 standard reflections every 97 reflections
5407 independent reflections	intensity decay: 1%

### (II) 1,3-Dibromo-2-iodo-5-nitrobenzene Refinement

**Table:**

Refinement on *F*^2^	Secondary atom site location: difference Fourier map
Least-squares matrix: full	Hydrogen site location: inferred from neighbouring sites
*R*[*F*^2^ > 2σ(*F*^2^)] = 0.061	H-atom parameters constrained
*wR*(*F*^2^) = 0.153	*w* = 1/[σ^2^(*F*_o_^2^) + (0.053*P*)^2^] where *P* = (*F*_o_^2^ + 2*F*_c_^2^)/3
*S* = 0.95	(Δ/σ)_max_ < 0.001
5407 reflections	Δρ_max_ = 0.84 e Å^−^^3^
218 parameters	Δρ_min_ = −0.84 e Å^−^^3^
0 restraints	Extinction correction: SHELXL2014 (Sheldrick, 2015), Fc^*^=kFc[1+0.001xFc^2^λ^3^/sin(2θ)]^-1/4^
0 constraints	Extinction coefficient: 0.00093 (11)
Primary atom site location: structure-invariant direct methods	

### (II) 1,3-Dibromo-2-iodo-5-nitrobenzene Fractional atomic coordinates and isotropic or equivalent isotropic displacement parameters (Å^2^)

**Table:**

	*x*	*y*	*z*	*U*_iso_*/*U*_eq_	
Br1	0.30362 (13)	0.43988 (8)	0.2401 (2)	0.0615 (4)	
I2	0.54556 (10)	0.36925 (4)	0.26460 (13)	0.0583 (3)	
Br3	0.73278 (12)	0.49074 (7)	0.26614 (18)	0.0561 (4)	
C1	0.4175 (11)	0.4967 (7)	0.2453 (16)	0.035 (3)	
C2	0.5191 (11)	0.4723 (6)	0.2535 (13)	0.033 (3)	
C3	0.5960 (11)	0.5182 (6)	0.2557 (17)	0.038 (3)	
C4	0.5754 (12)	0.5862 (6)	0.2478 (17)	0.040 (3)	
H4A	0.6264	0.6171	0.2474	0.048*	
C5	0.4763 (13)	0.6050 (7)	0.2405 (15)	0.046 (4)	
C6	0.3965 (12)	0.5642 (7)	0.2397 (18)	0.048 (4)	
H6A	0.3307	0.5809	0.2355	0.057*	
N1	0.4555 (13)	0.6807 (6)	0.2380 (17)	0.065 (3)	
O1	0.5145 (11)	0.7161 (5)	0.2088 (17)	0.085 (4)	
O2	0.3795 (15)	0.6989 (5)	0.2557 (18)	0.105 (4)	
Br11	−0.18918 (13)	0.30721 (8)	0.2488 (2)	0.0620 (4)	
I12	0.04866 (10)	0.37922 (4)	0.26639 (13)	0.0593 (3)	
Br13	0.24427 (12)	0.25893 (8)	0.2825 (2)	0.0588 (4)	
C11	−0.0736 (11)	0.2509 (7)	0.2562 (17)	0.039 (3)	
C12	0.0237 (12)	0.2769 (7)	0.2619 (14)	0.035 (3)	
C13	0.1046 (11)	0.2312 (6)	0.2672 (18)	0.039 (3)	
C14	0.0869 (13)	0.1639 (6)	0.2665 (18)	0.045 (4)	
H14A	0.1429	0.1344	0.2736	0.054*	
C15	−0.0137 (13)	0.1391 (8)	0.2553 (16)	0.045 (4)	
C16	−0.0922 (13)	0.1829 (6)	0.2507 (18)	0.043 (4)	
H16A	−0.1596	0.1668	0.2437	0.052*	
N11	−0.0302 (15)	0.0681 (6)	0.2483 (17)	0.066 (4)	
O11	0.0350 (12)	0.0323 (6)	0.2418 (18)	0.099 (4)	
O12	−0.1106 (14)	0.0493 (6)	0.2609 (17)	0.099 (4)	

### (II) 1,3-Dibromo-2-iodo-5-nitrobenzene Atomic displacement parameters (Å^2^)

**Table:**

	*U*^11^	*U*^22^	*U*^33^	*U*^12^	*U*^13^	*U*^23^
Br1	0.0558 (8)	0.0680 (9)	0.0684 (9)	−0.0167 (7)	0.0437 (7)	−0.0035 (7)
I2	0.0737 (6)	0.0268 (4)	0.0723 (7)	0.0048 (5)	0.0464 (5)	0.0017 (4)
Br3	0.0484 (7)	0.0636 (9)	0.0654 (9)	0.0047 (7)	0.0409 (7)	0.0002 (7)
C1	0.041 (7)	0.035 (7)	0.033 (6)	−0.012 (6)	0.027 (5)	−0.007 (5)
C2	0.043 (7)	0.017 (6)	0.035 (8)	0.002 (5)	0.024 (6)	0.000 (4)
C3	0.042 (6)	0.033 (7)	0.041 (7)	0.003 (5)	0.028 (6)	−0.004 (5)
C4	0.037 (7)	0.036 (8)	0.048 (8)	−0.005 (6)	0.027 (6)	−0.003 (6)
C5	0.046 (7)	0.023 (7)	0.040 (8)	0.005 (6)	0.015 (6)	−0.002 (5)
C6	0.036 (7)	0.064 (10)	0.045 (8)	−0.003 (7)	0.027 (6)	−0.016 (7)
N1	0.066 (8)	0.031 (7)	0.075 (9)	0.010 (7)	0.036 (7)	−0.001 (6)
O1	0.091 (8)	0.030 (6)	0.108 (9)	−0.003 (6)	0.053 (7)	0.003 (6)
O2	0.163 (12)	0.050 (7)	0.137 (11)	0.050 (8)	0.112 (10)	0.016 (6)
Br11	0.0522 (8)	0.0681 (9)	0.0685 (9)	0.0108 (7)	0.0404 (7)	−0.0033 (7)
I12	0.0755 (6)	0.0283 (4)	0.0735 (7)	−0.0069 (5)	0.0479 (5)	−0.0038 (4)
Br13	0.0488 (7)	0.0631 (9)	0.0703 (9)	−0.0088 (7)	0.0412 (7)	−0.0014 (7)
C11	0.043 (7)	0.035 (7)	0.041 (7)	−0.002 (6)	0.028 (6)	0.002 (6)
C12	0.041 (7)	0.033 (8)	0.039 (8)	0.007 (6)	0.030 (6)	0.004 (4)
C13	0.033 (6)	0.037 (8)	0.042 (7)	−0.005 (5)	0.022 (6)	−0.005 (6)
C14	0.051 (8)	0.029 (7)	0.044 (8)	0.008 (6)	0.025 (6)	0.000 (6)
C15	0.063 (9)	0.028 (7)	0.056 (9)	−0.015 (6)	0.045 (8)	−0.006 (5)
C16	0.046 (7)	0.031 (7)	0.042 (7)	−0.021 (6)	0.024 (6)	−0.015 (5)
N11	0.096 (10)	0.034 (7)	0.073 (9)	−0.018 (7)	0.057 (8)	−0.006 (6)
O11	0.101 (9)	0.029 (6)	0.147 (11)	−0.010 (6)	0.072 (8)	−0.005 (7)
O12	0.144 (11)	0.061 (7)	0.130 (10)	−0.042 (8)	0.106 (9)	−0.012 (6)

### (II) 1,3-Dibromo-2-iodo-5-nitrobenzene Geometric parameters (Å, º)

**Table:**

Br1—C1	1.894 (12)	Br11—C11	1.895 (14)
I2—C2	2.088 (12)	I12—C12	2.074 (14)
Br3—C3	1.875 (13)	Br13—C13	1.888 (13)
C1—C6	1.375 (18)	C11—C12	1.387 (17)
C1—C2	1.416 (17)	C11—C16	1.381 (18)
C2—C3	1.380 (17)	C12—C13	1.405 (17)
C3—C4	1.384 (16)	C13—C14	1.370 (16)
C4—C5	1.353 (18)	C14—C15	1.390 (19)
C4—H4A	0.9300	C14—H14A	0.9300
C5—C6	1.35 (2)	C15—C16	1.359 (19)
C5—N1	1.539 (18)	C15—N11	1.435 (19)
C6—H6A	0.9300	C16—H16A	0.9300
N1—O2	1.199 (17)	N11—O11	1.168 (19)
N1—O1	1.221 (19)	N11—O12	1.226 (18)
			
C6—C1—C2	120.8 (13)	C12—C11—C16	121.2 (14)
C6—C1—Br1	116.4 (11)	C12—C11—Br11	121.4 (11)
C2—C1—Br1	122.8 (10)	C16—C11—Br11	117.3 (11)
C3—C2—C1	118.1 (12)	C11—C12—C13	117.3 (13)
C3—C2—I2	123.6 (10)	C11—C12—I12	120.7 (11)
C1—C2—I2	118.4 (10)	C13—C12—I12	122.0 (10)
C2—C3—C4	122.0 (13)	C14—C13—C12	120.8 (13)
C2—C3—Br3	121.2 (10)	C14—C13—Br13	117.0 (11)
C4—C3—Br3	116.8 (11)	C12—C13—Br13	122.2 (10)
C5—C4—C3	115.9 (13)	C13—C14—C15	120.8 (14)
C5—C4—H4A	122.0	C13—C14—H14A	119.6
C3—C4—H4A	122.0	C15—C14—H14A	119.6
C4—C5—C6	126.6 (14)	C16—C15—C14	118.8 (14)
C4—C5—N1	116.2 (15)	C16—C15—N11	122.8 (14)
C6—C5—N1	117.2 (15)	C14—C15—N11	118.3 (15)
C5—C6—C1	116.7 (13)	C15—C16—C11	121.0 (14)
C5—C6—H6A	121.7	C15—C16—H16A	119.5
C1—C6—H6A	121.7	C11—C16—H16A	119.5
O2—N1—O1	126.4 (14)	O11—N11—O12	124.2 (16)
O2—N1—C5	117.6 (14)	O11—N11—C15	120.6 (18)
O1—N1—C5	115.9 (17)	O12—N11—C15	115.0 (15)
			
C6—C1—C2—C3	0.0 (16)	C16—C11—C12—C13	−1.7 (16)
Br1—C1—C2—C3	179.7 (9)	Br11—C11—C12—C13	180.0 (9)
C6—C1—C2—I2	179.4 (9)	C16—C11—C12—I12	179.2 (9)
Br1—C1—C2—I2	−1.0 (12)	Br11—C11—C12—I12	0.8 (13)
C1—C2—C3—C4	−0.8 (17)	C11—C12—C13—C14	0.1 (17)
I2—C2—C3—C4	179.9 (9)	I12—C12—C13—C14	179.2 (9)
C1—C2—C3—Br3	−179.9 (9)	C11—C12—C13—Br13	−178.2 (9)
I2—C2—C3—Br3	0.7 (14)	I12—C12—C13—Br13	0.9 (14)
C2—C3—C4—C5	0.9 (18)	C12—C13—C14—C15	1.7 (19)
Br3—C3—C4—C5	−179.9 (9)	Br13—C13—C14—C15	−179.9 (10)
C3—C4—C5—C6	−0.2 (18)	C13—C14—C15—C16	−1.9 (18)
C3—C4—C5—N1	178.2 (11)	C13—C14—C15—N11	177.8 (12)
C4—C5—C6—C1	−0.5 (19)	C14—C15—C16—C11	0.3 (18)
N1—C5—C6—C1	−178.9 (10)	N11—C15—C16—C11	−179.5 (12)
C2—C1—C6—C5	0.6 (17)	C12—C11—C16—C15	1.6 (19)
Br1—C1—C6—C5	−179.1 (9)	Br11—C11—C16—C15	180.0 (10)
C4—C5—N1—O2	−171.0 (13)	C16—C15—N11—O11	175.7 (14)
C6—C5—N1—O2	7.6 (18)	C14—C15—N11—O11	−4 (2)
C4—C5—N1—O1	12.4 (17)	C16—C15—N11—O12	−8.3 (19)
C6—C5—N1—O1	−169.1 (12)	C14—C15—N11—O12	171.9 (13)
